# Impact of Patient Income and Insurance on Postoperative Mortality After Total Pancreatectomy for Pancreatic Neoplasms

**DOI:** 10.1002/jso.70062

**Published:** 2025-08-06

**Authors:** Gracia Maria Vargas, Mohammad Saad Farooq, Neha Shafique, Major Kenneth Lee, Charles M. Vollmer, John T. Miura, Giorgos C. Karakousis

**Affiliations:** ^1^ Department of Surgery Hospital of the University of Pennsylvania Philadelphia Pennsylvania USA

**Keywords:** disparities, pancreatic cancer, socioeconomic status, surgical outcomes, total pancreatectomy

## Abstract

**Background:**

Total pancreatectomies (TP) are rare high‐risk operations requiring complex postoperative management. Hospital factors are known to impact pancreatectomy outcomes, but the role of patient socioeconomic status on TP outcomes remains poorly understood. This retrospective study assesses the impact of income and insurance on 90‐day mortality after TP.

**Methods:**

Adults (≥ 18 years) who underwent TP for pancreatic neoplasms were identified in the National Cancer Database (2008–2022). Kaplan–Meier analysis assessed 90‐day survival stratified by income and insurance. Univariable and multivariable Cox proportional hazards analyses were performed. Multivariable Cox models adjusting for clinical, oncologic, and facility characteristics assessed the impact of income and insurance on postoperative survival.

**Results:**

Among 11 321 patients, 90‐day mortality was 8.0%. Facility volume and Commission on Cancer designation were associated with 90‐day mortality (*p* < 0.001), as were patient insurance and income (*p* < 0.01). High‐volume facilities had more male, non‐Hispanic White, privately insured, and high‐income patients than low‐volume facilities (*p* < 0.001). Ninety‐day survival differed significantly between high‐ and lower‐income patients with each insurance type (*p* < 0.001). On adjusted Cox analysis, high income was associated with better 90‐day survival for patients with Medicaid or no insurance (HR 0.42, *p* = 0.049) and Medicare (HR 0.77, *p* = 0.008). Ninety‐day mortality did not differ between high‐ and lower‐income patients with private insurance (HR 1.09, *p* = 0.597). Private insurance was associated with better 90‐day survival versus patients with Medicaid or no insurance among lower‐income patients (HR 0.57, *p* = 0.002), but not high‐income patients (HR 1.43, *p* = 0.413).

**Conclusions:**

Patient insurance and income influence 90‐day mortality after TP, independent of clinical and facility factors. These findings suggest that financial burdens meaningfully impact postoperative recovery following TP, highlighting the need for careful preoperative screening and planning to ensure adequate support for at‐risk patients.

## 1 Introduction

Total pancreatectomy (TP) for malignancy is a rare operation that largely fell out of favor due to its high morbidity (30%–87%) and mortality (0%–23%) with limited evidence of oncologic benefit [1, 2, 3]. The postoperative course of TP is marked by the significant challenges of managing the resultant endocrine and exocrine insufficiency, which subject patients to lifelong medication burdens, decreased quality of life, and the possibility of hypoglycemia [4, 5]. Despite its drawbacks, there remain cases in which TP is a necessary procedure, including for resection of a confirmed or suspected pancreatic malignancy. Some TPs are planned electively, while others are an intraoperative decision based on the results of frozen section analysis [6, 7, 8]. While TPs remain rare, patients who require this operation in the modern era now benefit from improved delayed‐release formulations of pancrelipase, long‐acting insulin, and technologies like continuous glucose monitors and insulin pumps, which present significant opportunities to improve postoperative survival and quality of life after TP [6]. In this setting, there is evidence that high‐volume pancreatectomy centers now have morbidity and mortality rates for TP comparable to those of pancreaticoduodenectomy [9, 10].

There is a small body of evidence that suggests recent improvements in outcomes from TP, though many of these studies have come out of high‐volume academic institutions, which have been shown to have better postoperative pancreatectomy outcomes [11, 12, 13, 14, 15, 16, 17, 18, 19]. However, nearly half of patients who undergo TP for cancer have their operations at community cancer centers, posing the potential for wide variability in postoperative care, resources, and outcomes [16, 20, 21]. Multiple studies have found disparities on the basis of race, socioeconomic status, insurance status, gender, and age with respect to receipt of surgical resection and treatment at high‐volume facilities [22]. As a result, patients of lower socioeconomic status may be disproportionately exposed to the risks of undergoing complex operations like TPs at lower‐volume facilities. Whether socioeconomic disadvantage contributes independently to worse postoperative outcomes, beyond differences in treatment setting, remains unclear.

This study sought to examine the relationship between patient insurance status and income status and postoperative mortality following TP, independent of hospital factors. Ninety‐day mortality was chosen as the primary outcome of this study, in keeping with literature suggesting that for pancreas resections and cancer operations, this metric encompasses early operative complications, delayed postoperative deaths of critically ill patients, and medical management after discharge [23, 24, 25, 26].

## 2 Methods

### 2.1 Data Source

The National Cancer Database (NCDB) [27] is a hospital‐based cancer registry run jointly by the American College of Surgeons and the American Cancer Society encompassing data from over 1500 Commission on Cancer (CoC)‐accredited facilities in the United States. The cases in the NCDB represent ~70% of all patients newly diagnosed with cancer in the United States, and include patient and hospital characteristics, cancer diagnosis and staging, cancer treatment modalities, and timing of treatment [28]. The NCDB data are deidentified and compliant with the Health Insurance Portability and Accountability Act; this study was therefore exempt from review by the Institutional Review Board of the University of Pennsylvania.

### 2.2 Study Design and Population

This retrospective study used the NCDB Pancreas participant user file to identify patients ≥ 18 years of age who underwent TP with or without subtotal gastrectomy or duodenectomy for pancreatic neoplasms between 2008 and 2022. Patients who underwent surgery at a facility without consistent annual reporting to the NCDB within the study period were excluded to ensure accurate calculation of annual facility volume. The primary outcome of this study was 90‐day postoperative mortality after TP. Patient socioeconomic factors of interest were race, insurance status, median income quartile, rural residence, and distance to facility. Facility factors were CoC designation (“facility type”) and facility volume. High facility volume was defined as an average of ≥ 20 annual pancreatectomies of any kind and ≥ 2 annual TPs in the NCDB within the study period; these metrics were chosen based on existing literature identifying a 20‐case threshold for improved outcomes of major pancreatectomies, as well as the distribution of TPs in the facilities included in this cohort (Table 1) [11, 15, 29].

**Table 1:** Characteristics of NCDB facilities performing total pancreatectomies (2008–2022).

Facility characteristics	Low‐volume facilities *N* = 563 (88.2%)	High‐volume facilities *N* = 75 (11.8%)	*p*
Facility type			< 0.001
Community cancer program	44 (7.8)	6 (8.0)	
Comprehensive community cancer Program	248 (44.1)	0 (0)	
Academic/research program	130 (23.1)	58 (77.3)	
Integrated network cancer program	138 (24.5)	11 (14.7)	
Avg. annual pancreas resection volume, median, (IQR)	4.1 (1.4, 9.1)	34.8 (27.9, 46.9)	< 0.001
Avg. annual TP volume, median (IQR)	0.4 (0.13, 1.0)	3.8 (2.8, 5.3)	< 0.001

### 2.3 Statistical Analyses

Cohort descriptive statistics were performed using *χ* ^2^ tests for categorical variables and Wilcoxon rank sum tests for continuous variables. Kaplan–Meier survival analysis was performed for insurance and income subgroups. Univariable and multivariable Cox proportional hazards models were also performed. Multivariable Cox modeling assessed the impact of patient income on 90‐day survival stratified by insurance type, and the impact of insurance type on 90‐day survival stratified by income, controlling for the following predefined covariables: age, sex, Charlson–Deyo comorbidity score, TP type, tumor histology, primary tumor site, neoadjuvant chemotherapy, and neoadjuvant radiation therapy, facility volume, and facility type. All data analyses were conducted in Stata version 16.1 (StataCorp LLC; College Station, TX).

## 3 Results

### 3.1 Study Cohort

The final cohort included 11 321 patients (2008–2022) who underwent TP, with 6058 (53.5%) treated at low‐volume facilities and 5263 (46.5%) at high‐volume facilities (Table 2). Patients at high‐volume facilities were more likely to be male (*p* = 0.016), non‐Hispanic White (*p* < 0.001), have private insurance (*p* < 0.001), reside in a top income quartile zip code (*p* < 0.001), and travel greater distances for care (*p* < 0.001).

**Table 2:** Total pancreatectomy patient characteristics by facility volume status.

Patient characteristics	Low‐volume facility patients *N* = 6058 (53.5%)	High‐volume facility patients *N* = 5263 (46.5%)	*p*
Age, median (IQR)	66 (59, 73)	66 (58, 73)	0.077
Female	3003 (49.6)	2489 (47.3)	0.016
Race/ethnicity			< 0.001
Non‐hispanic white	4468 (73.8)	4288 (81.5)	
Hispanic white	449 (7.4)	235 (4.5)	
Black or African American	785 (13.0)	451 (8.6)	
Asian/pacific islander	203 (3.4)	161 (3.1)	
Other	153 (2.5)	128 (2.4)	
Insurance status			< 0.001
Uninsured	199 (3.3)	81 (1.5)	
Private insurance or managed care	2094 (34.6)	2044 (38.8)	
Medicaid	381 (6.3)	232 (4.4)	
Medicare	2,180 (52.5)	2,744 (52.1)	
Other government insurance	107 (1.8)	94 (1.8)	
Unknown insurance status	97 (1.6)	68 (1.3)	
Median income quartile (2020)			< 0.001
< $46 277	884 (17.1)	644 (14.1)	
$46 277–$57 856	1090 (21.1)	920 (20.2)	
$57 857–$74 062	1286 (24.9)	1118 (24.5)	
> $74 062	1912 (37.0)	1883 (41.3)	
Urban/rural residence			0.971
Metropolitan	4942 (84.1)	4073 (81.2)	
Urban	815 (13.9)	671 (13.9)	
Rural	118 (2.0)	94 (1.9)	
Distance to facility (miles), Median (IQR)	11.6 (5.2, 28.1)	31.1 (12.9, 81.4)	< 0.001
Charlson–Deyo Comorbidity Index			0.798
CDCI Score 0	3809 (62.9)	3347 (63.6)	
CDCI Score 1	1591 (26.3)	1361 (25.9)	
CDCI Score 2	428 (7.1)	352 (6.7)	
CDCI Score 3+	230 (3.8)	203 (3.9)	
Primary site			< 0.001
Pancreatic head or neck	3667 (60.5)	3376 (64.2)	
Pancreatic body	526 (8.7)	464 (8.8)	
Pancreatic tail	943 (15.6)	588 (11.2)	
Other	922 (15.2)	588 (11.2)	
Tumor histology			0.011
Pancreatic adenocarcinoma	4799 (79.2)	4167 (79.2)	
Neuroendocrine tumor	969 (16.0)	802 (15.2)	
Intraductal papillary mucinous neoplasm	202 (3.3)	232 (4.4)	
Other	88 (1.5)	62 (1.2)	
Procedure type			0.171
Pylorus‐preserving total pancreatectomy	2151 (35.5)	1804 (34.3)	
Classical total pancreatectomy	3907 (64.5)	3459 (65.7)	
Neoadjuvant radiation	301 (5.0)	584 (11.1)	< 0.001
Neoadjuvant chemotherapy	1094 (18.1)	1489 (28.3)	< 0.001

**Figure 1:**
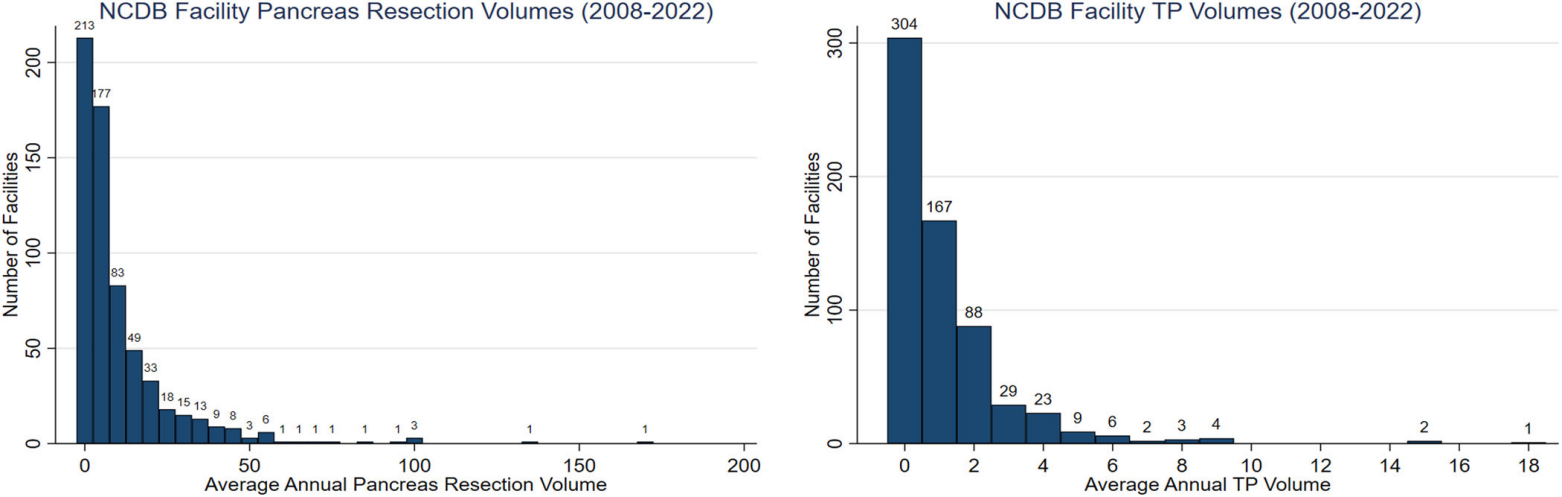
Annual pancreas resection volume and total pancreatectomy volume for NCDB facilities performing total pancreatectomies (2008–2022).

Patients in the study cohort underwent surgery at 638 unique facilities, of which 563 (88.2%) were low‐volume and 75 (11.8%) were high‐volume (Table 1). High‐volume facilities were predominantly academic/research centers (*p* < 0.001) with a median annual pancreas resection volume of 34.8, compared to 4.1 at low‐volume facilities (*p* < 0.001). Facility volume distribution was skewed; over half of the surgical facilities in this cohort performed fewer than five pancreatectomies of any kind and fewer than one TP annually (Figure 1).

Clinically, there was no difference in comorbidities between high‐ and low‐volume facility patients (*p* > 0.05, Table 2). A higher proportion of high‐volume facility patients had pancreatic head lesions, while a higher proportion of low‐volume facility patients had pancreatic tail lesions (*p* < 0.001). Tumor histologies differed marginally between low‐ and high‐volume facilities (*p* = 0.011); 79.2% were pancreatic adenocarcinoma in both low‐ and high‐volume facilities, while neuroendocrine tumors were more common in low‐volume facilities (16.0% vs. 15.2%) and intraductal papillary mucinous neoplasms were more common in high‐volume facilities (4.4% vs. 3.3%, *p* = 0.011). Patients treated at high‐volume facilities had higher rates of neoadjuvant radiation (*p* < 0.001) and neoadjuvant chemotherapy (*p* < 0.001). There was no difference in surgical approaches (classical vs. pylorus‐preserving TP) between high‐ and low‐volume facilities (*p* = 0.171).

### 3.2 Surgical Outcomes

Most surgical outcomes differed between low‐ and high‐volume facilities (Table 3). High‐volume facilities had significantly higher rates of R0 resection (83.4% vs. 80.5%), shorter surgical inpatient stay (IQR 6–11 vs. 6–13), lower mortality during the index surgical admission (7.8% vs. 9.8%, *p* < 0.001), lower 30‐day mortality (2.7% vs. 5.3%, *p* < 0.001), and lower 90‐day mortality (5.8% vs. 10.0%, *p* < 0.001) compared to low‐volume facilities. There was no difference in 30‐day readmission rates.

**Table 3:** Total pancreatectomy outcomes by facility volume status.

Surgical outcomes	Low‐volume facility patients *N* = 6058 (53.5%)	High‐volume facility patients *N* = 5263 (46.5%)	*p*
Resection margin			< 0.001
No residual tumor (R0 resection)	4874 (80.5)	4390 (83.4)	
Positive margins	1100 (18.2)	814 (15.5)	
Unknown or indeterminate margins	84 (1.4)	59 (1.1)	
Surgical inpatient stay, median (IQR)	8 (6, 13)	8 (6, 11)	< 0.001
Readmission within 30 days	492 (8.1)	416 (7.9)	0.646
Mortality during surgical admission	591 (9.8)	408 (7.8)	< 0.001
Mortality within 30 days	299 (5.3)	132 (2.7)	< 0.001
Mortality within 90 days	562 (10.0)	289 (5.8)	< 0.001

### 3.3 Unadjusted 90‐Day Survival Analysis

**Figure 2:**
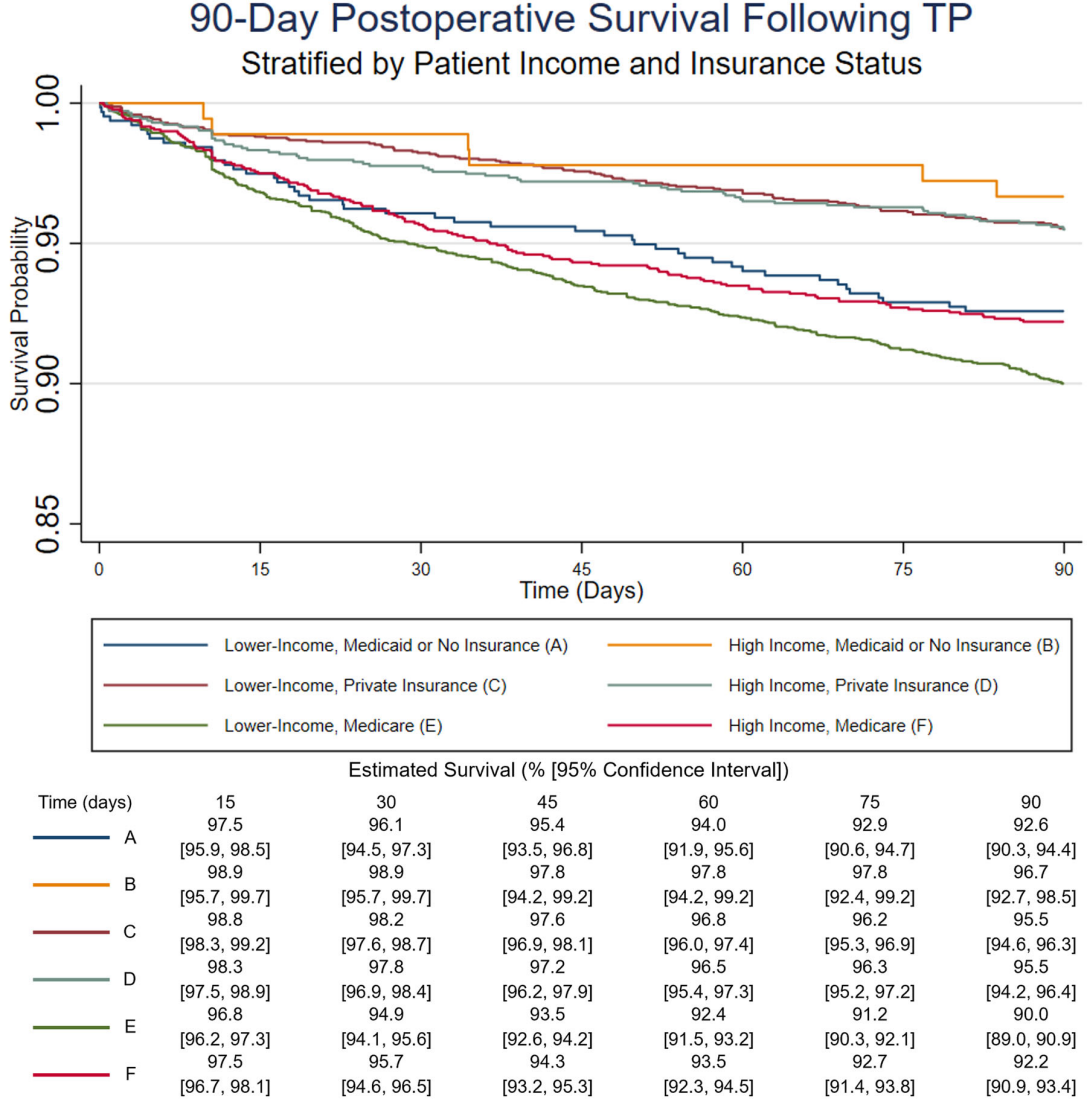
Kaplan–Meier analysis for 90‐day survival following total pancreatectomy (TP).

Kaplan–Meier analysis of 90‐day postoperative survival was stratified by patient income (where “lower‐income” represents the lower three income quartiles, and “high income” represents the top income quartile) and patient insurance status. Lower‐income patients with Medicare had the lowest 90‐day survival (90.0% survival, 95% confidence interval 89.0–90.9; Figure 2), followed by high‐income patients with Medicare (92.2% survival, 95% CI 90.9–93.4) and lower‐income patients with Medicaid or no insurance (92.6% survival, 95% CI 90.3–94.4). High‐income patients with Medicaid or no insurance (209 patients, 1.9% of the cohort) had the highest 90‐day survival (96.7%, 95% CI 92.7, 98.5). Privately insured patients had the second‐highest survival, which did not differ between lower‐ and high‐income patients (95.5% survival for both; 95% CI 94.6–96.3, and 94.2–96.4, respectively).

### 3.4 Adjusted 90‐Day Survival Analysis

On univariable Cox analysis (Table 4), high income (i.e., highest income quartile) was associated with better survival compared to lower income (HR 0.80, *p* = 0.004). Compared to patients with Medicaid or no insurance, those with private insurance or managed care had better survival (HR 0.68, *p* = 0.014), while those with Medicare had worse survival (HR 1.44, *p* = 0.011). Facility type, facility volume, age, Charlson–Deyo score, TP type, tumor histology, primary site, and neoadjuvant radiation status were also significantly associated with 90‐day survival (*p* < 0.05).

**Table 4:** Univariable Cox proportional hazards analysis for 90‐day postoperative survival.

Variable	Hazard ratio	95% confidence interval	*p*
High income (ref: lower income)	0.80	[0.681, 0.932]	0.004
Insurance type (ref: Medicaid or no insurance)			
Private insurance or managed care	0.68	[0.501, 0.927]	0.014
Medicare	1.44	[1.088, 1.916]	0.011
High volume facility (ref: low volume facility)	0.62	[0.534, 0.718]	< 0.001
Facility type (ref: community cancer program)			
Comprehensive community cancer program	0.54	[0.343, 0.858]	0.009
Academic/research program	0.35	[0.222, 0.547]	< 0.001
Integrated network cancer program	0.51	[0.324, 0.817]	0.005
Age (years)	1.04	[1.034, 1.049]	< 0.001
Charlson–Deyo score (ref: 0)			
1	1.02	[0.859, 1.200]	0.860
2	1.25	[0.964, 1.632]	0.092
3+	1.50	[1.083, 2.066]	0.015
Pylorus‐preserving TP (ref: classical TP)	0.63	[0.533, 0.739]	< 0.001
Histology (ref: pancreatic adenocarcinoma)			
Neuroendocrine tumor	0.38	[0.285, 0.500]	< 0.001
Intraductal papillary mucinous neoplasm	0.45	[0.270, 0.752]	0.002
Other	0.36	[0.136, 0.973]	0.044
Primary site (ref: pancreatic head or neck)			
Pancreatic body	0.65	[0.484, 0.874]	0.004
Pancreatic tail	0.59	[0.459, 0.763]	< 0.001
Other	0.86	[0.700, 1.050]	0.137
Neoadjuvant radiation	1.35	[1.070, 1.707]	0.011
Neoadjuvant chemotherapy	0.94	[0.789, 1.119]	0.486

Multivariable Cox analysis controlling for facility and patient clinical factors (Table 5a) found that patients with Medicaid or no insurance had better 90‐day survival if they had high income (HR 0.42, *p* = 0.049) and if they were treated at a high‐volume facility (HR 0.43, *p* = 0.020). Among patients with private insurance or managed care, there was no association between 90‐day mortality and high income (HR 1.09, *p* = 0.597) or high facility volume (HR 0.83, *p* = 0.309). Patients with Medicare had lower 90‐day mortality risk if they had high income (HR 0.77, *p* = 0.008) and if they were treated at a high‐volume facility (HR 0.70, *p* = 0.001). Among patients with lower income, those with Medicaid or no insurance had worse survival than those with private insurance (Table 5b; HR 0.57, *p* = 0.002), but no difference in survival compared to Medicare patients (HR 0.84, *p* = 0.313). In contrast, among those with high income, there was no difference in survival between those with Medicaid or no insurance compared to those with private insurance (HR 1.46, *p* = 0.375) and those with Medicare (HR 1.43, *p* = 0.413).

**Table 5a:** Multivariable Cox proportional hazards analysis for 90‐day postoperative survival, stratified by insurance type.

Variable	Medicaid or no insurance (*n* = 893)			Private insurance or managed care (*n* = 4138)			Medicare (*n* = 5924)		
	Hazard ratio	95% confidence interval	*p*	Hazard ratio	95% confidence interval	*p*	Hazard ratio	95% confidence interval	*p*
High income (ref: lower income)	0.42	[0.180, 0.996]	0.049	1.09	[0.797, 1.484]	0.597	0.77	[0.629, 0.931]	0.008
High volume facility (ref: low volume facility)	0.43	[0.209, 0.874]	0.020	0.83	[0.588, 1.183]	0.309	0.70	[0.566, 0.858]	0.001
Facility type (ref: community cancer program)									
Comprehensive community cancer program	1.18	[0.143, 9.664]	0.879	0.76	[0.234, 2.461]	0.645	0.56	[0.322, 0.968]	0.038
Academic/research program	0.96	[0.21, 7.652]	0.971	0.57	[0.173, 1.845]	0.345	0.43	[0.244, 0.744]	0.003
Integrated network cancer program	1.18	[0.141, 9.786]	0.881	0.77	[0.235, 2.534]	0.669	0.55	[0.316, 0.965]	0.037
Age (years)	1.01	[0.975, 1.039]	0.682	1.03	[1.017, 1.053]	< 0.001	1.04	[1.024, 1.051]	< 0.001
Charlson–Deyo score (ref: 0)									
1	0.90	[0.466, 1.723]	0.742	0.80	[0.549, 1.170]	0.252	1.01	[0.822, 1.233]	0.947
2	1.51	[0.510, 4.464]	0.457	1.24	[0.665, 2.305]	0.501	0.98	[0.713, 1.340]	0.886
3+	0.40	[0.526, 2.898]	0.358	1.61	[0.782, 3.296]	0.197	1.30	[0.883, 1.900]	0.185
Pylorus‐preserving TP (ref: classical TP)	1.02	[0.512, 2.031]	0.956	0.74	[0.507, 1.087]	0.126	0.73	[0.581, 0.912]	0.006
Histology (ref: pancreatic adenocarcinoma)									
Neuroendocrine tumor	0.39	[0.131, 1.162]	0.091	0.41	[0.233, 0.715]	0.002	0.61	[0.414, 0.892]	0.011
Intraductal papillary mucinous neoplasm	0.94	[0.126, 7.080]	0.954	0.15	[0.021, 1.082]	0.060	0.48	[0.268, 0.854]	0.013
Other	< 0.001	—	1.000	< 0.001	—	1.000	1.49	[0.552, 4.015]	0.432
Primary site (ref: head or neck)									
Pancreatic body	0.96	[0.312, 2.925]	0.937	1.04	[0.567, 1.911]	0.897	0.78	[0.531, 1.158]	0.221
Pancreatic tail	0.89	[0.313, 2.507]	0.819	1.11	[0.642, 1.933]	0.700	0.723	[0.512, 1.022]	0.066
Other	0.44	[0.153, 1.274]	0.131	< 0.001	—	1.000	1.16	[0.896, 1.492]	0.264
Neoadjuvant radiation	1.56	[0.514, 4.715]	0.433	2.07	[1.162, 3.692]	0.014	2.00	[1.368, 2.937]	< 0.001
Neoadjuvant chemotherapy	0.82	[0.351, 1.904]	0.640	0.62	[0.391, 0.992]	0.046	0.74	[0.559, 0.992]	0.044

**Table 5b:** Multivariable Cox proportional hazards analysis for 90‐day postoperative survival, stratified by income.

Variable	Lower income (*n* = 7526)			High income (*n* = 3795)		
	Hazard ratio	95% confidence interval	*p*	Hazard ratio	95% confidence interval	*p*
Insurance type (ref: Medicaid or no insurance)						
Private insurance or managed care	0.57	[0.407, 0.810]	0.002	1.46	[0.632, 3.388]	0.375
Medicare	0.84	[0.596, 1.180]	0.313	1.43	[0.609, 3.348]	0.413
High volume facility (ref: low volume facility)	0.74	[0.604, 0.904]	0.003	0.62	[0.442, 0.856]	0.004
Facility type (ref: Community Cancer Program)						
Comprehensive Community Cancer Program	0.57	[0.326, 0.981]	0.043	0.64	[0.230, 1.759]	0.384
Academic/Research Program	0.46	[0.265, 0.801]	0.006	0.43	[0.155, 1.203]	0.108
Integrated Network Cancer Program	0.61	[0.349, 1.064]	0.082	0.49	[0.174, 1.381]	0.177
Age (years)	1.03	[1.022, 1.046]	< 0.001	1.03	[1.013, 1.051]	0.001
Charlson–Deyo score (ref: 0)						
1	1.03	[0.846, 1.255]	0.765	0.72	[0.503, 1.019]	0.064
2	0.85	[0.606, 1.203]	0.366	1.64	[1.047, 2.566]	0.031
3+	1.24	[0.845, 1.813]	0.274	1.42	[0.721, 2.801]	0.310
Pylorus‐preserving TP (ref: classical TP)	0.71	[0.563, 0.885]	0.003	0.82	[0.592, 1.148]	0.252
Histology (ref: pancreatic adenocarcinoma)						
Neuroendocrine tumor	0.63	[0.450, 0.890]	0.009	0.32	[0.158, 0.628]	0.001
Intraductal papillary mucinous neoplasm	0.45	[0.238, 0.842]	0.013	0.37	[0.136, 1.003]	0.051
Other	1.14	[0.416, 3.128]	0.798	< 0.001	—	1.000
Primary site (ref: head or neck)						
Pancreatic body	0.86	[0.591, 1.249]	0.427	0.86	[0.481, 1.526]	0.599
Pancreatic tail	0.83	[0.592, 1.150]	0.256	0.81	[0.473, 1.382]	0.438
Other	0.99	[0.762, 1.289]	0.946	1.35	[0.933, 1.956]	0.112
Neoadjuvant radiation	1.85	[1.281, 2.658]	0.001	2.36	[1.349, 4.119]	0.003
Neoadjuvant chemotherapy	0.73	[0.552, 0.953]	0.021	0.69	[0.441, 1.078]	0.103

## 4 Discussion

This study found that patient income and insurance status both significantly impact 90‐day mortality following TP in a large national cohort. High income and private insurance were both associated with better 90‐day postoperative survival (Table 4). Within insurance groups, high income was associated with better 90‐day survival among patients with Medicaid or no insurance and those with Medicare; in contrast, there was no difference in survival between lower‐income and high‐income individuals with private insurance (Figure 2 and Table 5a). Further, insurance type had a significant impact on survival among low‐income individuals, but not high‐income individuals (Table 5b). These findings suggest that the financial burdens of postoperative recovery from TP may contribute to mortality within the first 90 days of surgery, and further, that private insurance may mitigate the mortality risk associated with lower income after this high‐risk operation.

Centralization of care to high‐volume academic centers has been proposed to improve outcomes and costs for complex procedures like TP, which are particularly volume‐sensitive [30, 31, 32]. However, racial and socioeconomic disparities exist in patient access to surgery, neoadjuvant and adjuvant therapies, and treatment at high‐volume centers for pancreatic cancer [22, 33, 34, 35, 36, 37]. A recent California study found that 70% of Medicare patients undergoing pancreatectomy lacked access to high‐volume hospitals due to insurance restrictions [38]. This study builds on that literature by demonstrating that patient insurance and income impact postoperative mortality following TP independent of disparities in access to high‐volume cancer centers.

Apart from their inherent surgical risk and complexity, TPs impart significant endocrine and exocrine insufficiency that must be managed indefinitely after surgery, causing significant challenges for patients to manage [39]. Insulin‐dependent diabetes following TP (type 3c diabetes mellitus) is particularly difficult to manage due to rapid changes between hyper‐ and hypoglycemia, exacerbated by rapid intestinal transit (diarrhea) in the absence of pancreatic enzymes [40]. High‐volume facilities may have more available resources to support patients through these challenges, for example, subspecialty consultant services while inpatient, and social services and close follow‐up after discharge [41, 42], however, our findings suggest that treatment at a high‐volume facility does not sufficiently mitigate the risks associated with low income or nonprivate insurance for patients undergoing TP. Though the financial burden of post‐TP diabetes has not been studied directly, 1 out of 20 patients in a small study of post‐TP patients had to change jobs to facilitate their diabetes management [39]—a notable finding given that most health insurance in the United States is obtained through the workplace [43].

While patient income is not a modifiable factor in the perioperative setting, this study's findings raise the question of whether optimizing insurance coverage—a potentially more feasible intervention—could improve outcomes including postoperative survival following TP. There is evidence that financial strain may influence the survival of cancer patients—for example, experiencing bankruptcy has been associated with significantly higher mortality across all malignancies (HR 1.86, *p* < 0.001) [43]. In contrast, private insurance has been associated with a significantly lower rate of 30‐day readmission post‐TP [18], and out‐of‐pocket payment (largely attributable to private insurance) has been associated with significantly higher overall survival after pancreatic cancer resection compared to no out‐of‐pocket payment (largely Medicare), with a median survival of 54 months compared to 28 months (*p* = 0.033) [44]. Despite evidence that patient income and insurance status matter for cancer surgery outcomes, documentation of these risk factors is poor. One study found that patients whose electronic health records were missing risk factors including a marker of financial status had significantly higher complication rates after elective cancer resections (49% vs. 24%, *p* = 0.021) [45], suggesting that targeted preoperative screening may present an opportunity for interventions to improve surgical outcomes. Routine screening for financial risk factors has been demonstrated to be feasible and effective in the outpatient setting at various cancer centers [46, 47, 48], presenting an opportunity to expand screening to include insurance needs. Although targeted interventions to optimize insurance prior to pancreatic resection have not been reported, a recent institutional study of patients admitted to the hospital via the emergency department found that those who gained insurance through a hospital‐based linkage program underwent significantly more elective procedures within 1 year of that admission than those who remained uninsured [49]. Future efforts to improve TP outcomes should consider incorporating a similar preoperative insurance screening and linkage program to identify and support at‐risk patients.

This study has several limitations. Its retrospective design and reliance on NCDB data limit the ability to capture all TP cases nationwide, as the NCDB includes only CoC‐accredited facilities and excludes cases for nonmalignant indications, such as chronic pancreatitis. Consequently, pancreatectomy volumes may be underestimated due to incomplete reporting. Additionally, this study was unable to evaluate surgeon volume, a factor known to account for a substantial proportion of the effect of hospital volume on pancreatic surgery outcomes [50, 51]. Finally, although the adjusted Cox analysis accounted for patient illness, Medicare beneficiaries may have greater underlying medical complexity than privately insured patients, which may not have been fully captured in the model. Despite these limitations, this study provides novel evidence that patient income and insurance status significantly impact 90‐day mortality after TP.

## 5 Conclusion

High income and private insurance were associated with significantly lower postoperative mortality following TP. While lower‐income patients generally had higher 90‐day mortality compared to high‐income patients, this difference did not exist among patients with private insurance. These findings suggest that preoperative optimization of insurance coverage may reduce postoperative mortality risk for low‐income patients undergoing TP. Surgeons should consider mechanisms to screen for at‐risk patients and connect them with insurance linkage and financial support programs prior to TP when possible.

## Conflicts of Interest

The National Cancer Database (NCDB) is a joint project of the Commission on Cancer (CoC) of the American College of Surgeons and the American Cancer Society. The CoC's NCDB and the hospitals participating in the CoC's NCDB are the source of the deidentified data used herein; they have not verified and are not responsible for the statistical validity of the data analysis or the conclusions derived by the authors. The other authors declare no conflicts of interest.

## Synopsis

This retrospective study used the National Cancer Database to assess the impact of patient insurance and income on 90‐day mortality after total pancreatectomy for pancreatic neoplasms. After controlling for patient and facility factors, private insurance and high income were both associated with significantly lower postoperative mortality.

## Data Availability

The data that support the findings of this study are available from the American College of Surgeons Commission on Cancer (CoC). Restrictions apply to the availability of these data, which were used under license for this study. NCDB data are available to investigators associated with CoC‐accredited cancer programs.
